# Recycling rigid polypropylene from mixed waste: Does the origin affect mechanical recyclate quality?

**DOI:** 10.1177/0734242X251357137

**Published:** 2025-08-20

**Authors:** Anna-Maria Lipp, Jessica Schlossnikl, Isabelle Gentgen, Thomas Koch, Vasiliki‑Maria Archodoulaki, Jakob Lederer

**Affiliations:** 1 Christian Doppler Laboratory for a Recycling-Based Circular Economy, Institute of Chemical, Environmental and Bioscience Engineering, TU Wien, Vienna, Austria; 2 Institute of Materials Science and Technology, Faculty of Mechanical and Industrial Engineering, TU Wien, Vienna, Austria; 3 Institute for Microtechnology and Photonics, Hochschule OST (Eastern Switzerland University of Applied Sciences), Buchs SG, Switzerland

**Keywords:** Plastic, packaging, recycling, municipal solid waste, circular economy

## Abstract

The increasing demand for plastic and the shortcoming of overall plastic recycling rates underscore the necessary transition towards a circular economy. In Austria, more than half of the generated plastic waste, especially non-packaging waste, is incinerated because of its disposal in mixed wastes. This highlights a vast untapped potential for recycling. While prior research has primarily focused on recycling plastic packaging waste from mixed waste origin, this study addresses a critical gap by exploring the recycling of non-packaging plastics in different concentrations by specifically examining rigid polypropylene (PP) sourced from a mixed waste material recovery facility. The methodology involves mechanical pre-processing of plastics, such as washing and sink-float separation, followed by polymer processing to evaluate the recyclates’ tensile (impact), thermal, morphological and rheological properties. Results indicate that the dirt content of rigid PP after sorting is comparable to that of separately collected waste. Furthermore, homogeneous recyclates with minor polyethene impurities were produced, the quality of which is comparable to commercially available recyclates regarding elastic modulus and yield stress. Although further research on odour contamination, substances of concern, long-term applicability and environmental and economic aspects is necessary, this study demonstrates that a substantial amount of PP can be recovered from mixed wastes in Austria. Ultimately, recycling such plastics can considerably contribute to circularity efforts.

## Introduction

The global demand for plastics continues to grow and can be predicted to rise even more (Plastics Europe, 2022; Williams and Rangel-Buitrago, 2022). By 2050, global plastic production is projected to reach between approximately 880 and 980 Mt (Dokl et al., 2024; Geyer et al., 2017; OECD, 2022), representing an increase of 80-135% compared to 2023 levels (OECD, 2022; Plastics Europe, 2024). The predominant linear nature of plastic production and disposal poses significant environmental, social and resource challenges (MacLeod et al., 2021; Stoett et al., 2024; Thompson et al., 2009). The largest portion of plastic waste is either incinerated, sent to landfills or improperly disposed of, that is, littered or openly dumped (Dokl et al., 2024; Ragossnig and Agamuthu, 2021). Recycling rates remain low, with post-consumer recyclates covering only 10% of the European plastic demand (Plastics Europe, 2022), leading to an ongoing reliance on virgin plastic materials. This imbalance between consumption and recycling underscores the need for waste management and recovery improvement. Although other measures, such as waste reduction or reuse, should be prioritised (Potting et al., 2017), recycling remains essential. Due to quality and efficiency aspects, separately collected material should generally be preferred for recycling (Antonopoulos et al., 2021; Lederer et al., 2022). However, the scope of the separate collection is limited, as (1) the collection rates for plastic packaging seem to have reached saturation, especially in urban centres, due to various logistical and socio-economic reasons (Feil et al., 2017; Schuch et al., 2023), and (2) many non-packaging plastics are to be disposed of properly via waste streams for incineration.

Austria exemplifies this challenge: in 2022, around 54 wt.% of plastic waste – equivalent to approximately 430,000 t annually – was disposed of in mixed waste streams, encompassing mixed municipal solid waste (MSW), commercial and bulky waste (BMK, 2024). About 95 wt.% of this waste is incinerated (BMK, 2024), leaving a major untapped potential for recycling. Despite its acute environmental and resource management implications, the recovery and recycling of such plastics from mixed waste are not practised in Austria.

Nevertheless, the recycling of plastics from mixed waste origin is often dismissed outright by both industry and policymakers. Commonly mentioned barriers include possible contaminations, economic viability, energy consumption and quality issues (Cimpan et al., 2015; Picuno et al., 2021; WKO, 2020). This has led to a self-reinforcing cycle of inaction, where the lack of information is used to justify the absence of investments, policy incentives, and even research, although the recovery is being successfully performed in other European Union (EU) countries (Cimpan et al., 2015; Montejo et al., 2013; Picuno et al., 2021; (Thanos) Bourtsalas and Themelis, 2022). As a result, the feasibility of recycling plastics from mixed fractions remains largely unexplored.

Yet, there is evidence that such recycling is viable with conventional processes and good processability (Möllnitz et al., 2021b). Qualities comparable with separate collection can be achieved, albeit with higher treatment effort (Luijsterburg and Goossens, 2014), and recovered fractions from mixed MSW are usually denoted by a lower price (Thoden van Velzen et al., 2017). However, it should be noted that there is often a lack of viable markets or recyclers willing to accept specific sorted plastic streams – whether derived from separate collection or recovered from mixed MSW – as the profit margin is very low, resulting in incineration unless supported by targeted policy intervention (Larrain et al., 2021).

Previous research investigated critical properties of mixed MSW-sourced plastic packaging, such as recycling yields and efficiencies, mechanical properties of the recyclate, molecular contamination and odour (Cabanes et al., 2020; Luijsterburg and Goossens, 2014; Thoden van Velzen et al., 2017, 2021a, 2021b). Thereby, recovered fractions, such as low densitiy polyethene (LDPE) bags from non-separately collected waste, are characterised by a much higher odour intensity (Cabanes et al., 2020). On the contrary, differences in the purity, mechanical properties and odour of separately collected and mixed MSW-sourced polyethene terephthalate (PET), polyethene (PE) and polypropylene (PP) recyclate have also been deemed relatively minor (Thoden van Velzen et al., 2021b). Nevertheless, it is suggested that high-quality PE can only be produced when the input material consists exclusively of a single grade of PE and without contamination from other polymers (Thoden van Velzen et al., 2021a), which is also not the case with separately collected packaging waste. Generally, quality aspects are highly context-dependent (Golkaram et al., 2022).

Most literature contributions focus on plastic packaging waste only (Lima et al., 2023), neglecting the vast non-packaging streams. Thus, the quality aspects of recycling, particularly regarding the impacts of non-packaging plastics from mixed waste, remain unknown. This study addresses this lacuna by focusing on recycling PP from mixed waste in Austria, emphasising the interplay between packaging and non-packaging plastics. Hence, rigid (non-)packaging plastic blends of mixed waste origin were washed, density separated and extruded to determine the quality parameters of the mechanical pre-processing as well as of the recyclate. By examining the critical aspects of recycled PP from mixed waste streams, this study aims to challenge entrenched perceptions of recyclate quality and provide actionable insights. In doing so, it offers a pathway to reduce incineration, increase recycling rates, and decrease dependence on virgin plastics.

## Materials and methods

### Materials: Sampling and sorting

This article further investigated material connected to former research; its properties, sampling methodology and conditions have been described in detail by Blasenbauer et al. (2024). The waste material was sampled from a mixed waste material recovery facility (MRF) located in Tyrol, Western Austria. The MRF treats MSW, as well as commercial and bulky waste. After shredding, the waste enters a screen, and the middle particle size fraction (40–250 mm) is directed to a ballistic separator, producing a 3D stream, dominated by hollow bodies, and a 2D stream, consisting mainly of flexibles. Subsequently, all fractions undergo metal separation (magnetic and non-magnetic). The investigated material was sourced from the 3D fraction, which is comprised of 24 wt.% plastic rigids (13 wt.% packaging and 11 wt.% non-packaging), 2 wt.% films and 18 wt.% compound plastics (Blasenbauer et al., 2024). Representative sampling was conducted at the end of a conveyor belt four times during 1 year to level out seasonal fluctuations, resulting in an annual mixed sample. All samples were air-dried. The contained rigid PP was manually sorted out according to their standard ASTM D7611/D7611M (2020) resin identification code, if applicable. Plastics without a code were identified with the help of Fourier transform infrared spectroscopy (FTIR Cary 630 spectrometer; Agilent Technologies, Santa Clara, CA, United States). The PP was divided into packaging and non-packaging following the categorisation of the Austrian Federal Ministry for Climate Action, Environment, Energy, Mobility, Innovation and Technology (BMK, n.d.), and then further split according to different visual aspects (white, coloured and transparent, non-coloured; referred to as natural in this article). Black plastics were put aside and not examined more closely.

**Figure 1.**
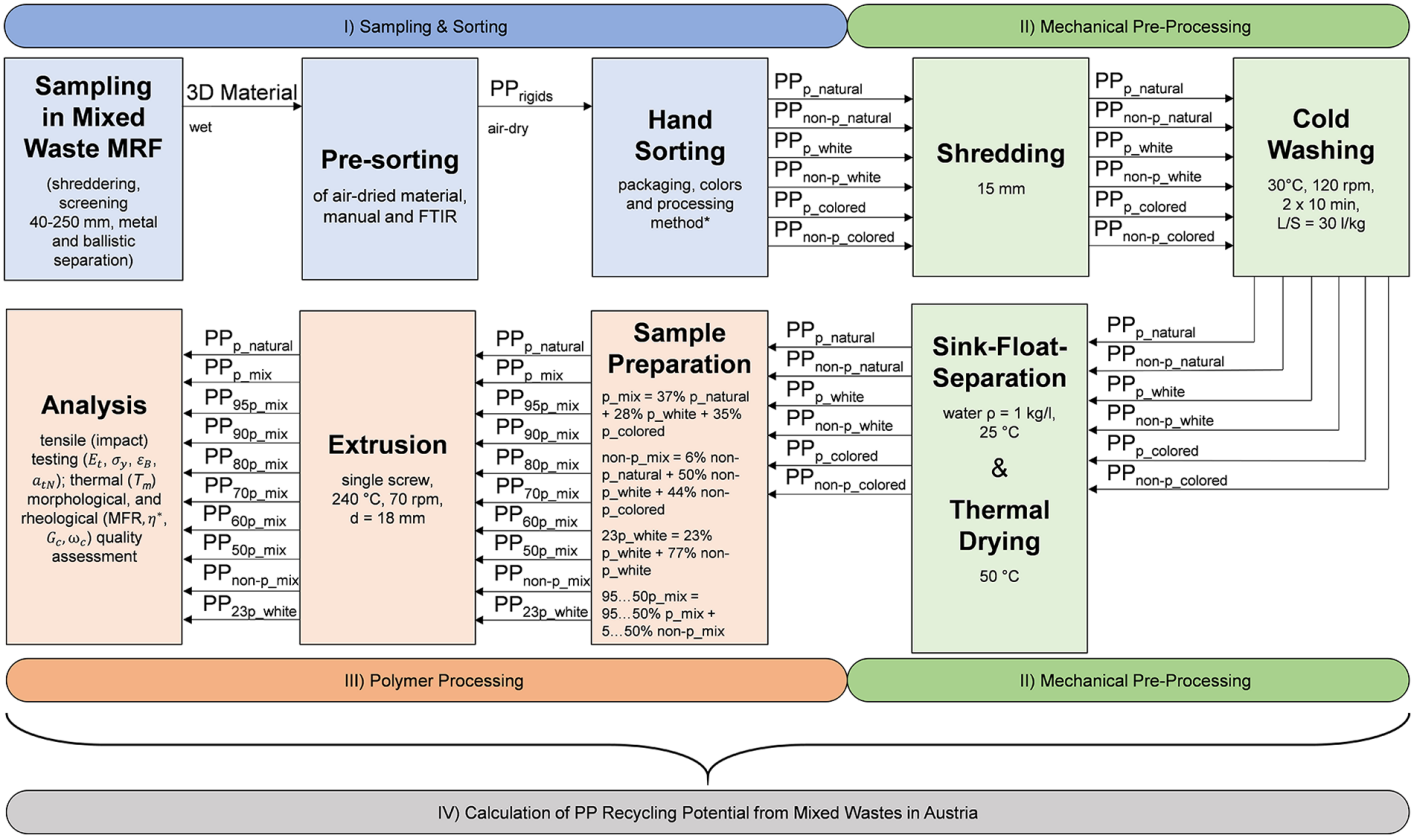
Methodological framework divided into three stages: (I) sampling and sorting, (II) mechanical pre-processing, (III) polymer processing and (IV) recycling potential calculation. FTIR: Fourier transform infrared spectroscopy; MRF: material recovery facility; PP: polypropylene.

To be able to draw better conclusions from the results, the packaging and non-packaging (of the respective colour category) were sorted manually according to their processing method (injection-moulded, blow-moulded, thermoformed). The allocation was decided visually, based on an injection moulding point, a pinch seam or the product itself; for example, buckets were always allocated to the injection moulding fraction, whereas smaller packaging trays without an injection moulding point are usually thermoformed. This allocation was complex in some cases due to the shredded and only partially complete (non-)packaging, so the allocation is only to be understood as a guideline; depictions of the sorted fractions can be found in the Supplemental Figure A1(a)–(r). Although this information was crucial to determine, the fractions were subsequently recombined and not treated separately (see Figure 1, indicated with an *).

### Mechanical pre-processing

#### Shredding, washing, sink-float-separation and drying

All PP fractions were individually shredded with a two-shaft shredder (RF137; Zakład Mechaniczny “PRECYZJA”, Czchów, Poland) and a round-hole screen insert of 15 mm screen size. Subsequently, each fraction was washed twice and in duplicate with the Vortex M6 (SDL Atlas, Rock Hill, SC, United States). Washing took place in water at 30°C for 10 min at 120 rpm agitation, with a liquid-to-solid ratio of 30 l·kg^−1^. No detergent was added in order to simulate a worst-case scenario representative of minimal cleaning conditions. To avoid the loss of smaller particles, a washing net was utilised. The wash water was collected and channelled through a filter to catch any dirt that had been washed off. The filter residue was dried and weighed.

Afterwards, all samples were submerged in a 75 l flotation tank filled with 25°C water for 30 min to separate non-PP particles, such as PET labels, with an intrinsic density ρ > 1000 kg·m^−3^. Then, the floating and sinking fractions were thermally dried at 50°C until mass stability was guaranteed and, finally, weighed.

#### Technical quality indicators

The objective of the washing process was to assess whether different colour groups of PP demonstrated variable levels of soiling. Furthermore, the sink-float separation was employed to determine non-washable foreign materials present within the samples. Thus, the foreign material content ( FMC ) on particle level after washing was calculated via equation (1).

(1) $${\mathrm{FMC}}_{\%}=\;\frac{m_{sinking}}{m_{sinking}+\;m_{floating}}\cdot 100\%$$

In addition, with equations (2) and (3), the dirt content ( DC ) of each sample based on dry matter was determined (Thoden van Velzen et al., 2017).

(2) $$m_{dissolved}=\;m_{feed}-m_{filter\_residue}-\;m_{sinking}-m_{floating}$$

(3) $${\mathrm{DC}}_{\%}=\;\frac{m_{filter\_residue}+m_{dissolved}}{m_{feed}}\cdot 100\%$$

Finally, the mass recovery rate of the product ( $R_{PP})$ was calculated following equation (4) (Thoden van Velzen et al., 2017).

(4) $$R_{PP}=\;\frac{m_{floating}}{m_{feed}}$$

### Polymer processing and analysing methods

#### Sample preparation of (non-)packaging blends

Following the initial separation, the packaging and non-packaging samples were recombined to form the following mixed-colour PP fractions: packaging mix (PP_p_mix_; as in the original stream composed of 37 wt.% natural, 35 wt.% coloured and 28 wt.% white packaging), non-packaging mix (PP_non-p_mix_; as in the original stream composed 6 wt.% natural, 44 wt.% coloured and 50 wt.% white non-packaging). Since technically no, or only a limited, separation in waste sorting processes between packaging and non-packaging is possible, these PP_p_mix_ and PP_non-p_mix_ fractions were utilised to prepare a concentration gradient series. This series entailed combinations of different increments of 95, 90, 80, 70, 60 and 50 wt.% PP_p_mix_ and the respective remaining fraction of PP_non-p_mix_. The primary goals were (1) to assess the extent to which the non-packaging fraction influences the material’s mechanical properties and (2) to map as many different modifications as possible of the rigid plastic waste flows that can occur in a mixed waste MRF. Additionally, a fraction (PP_23p_white_) containing 23 wt.% white packaging and 77 wt.% white non-packaging PP was created, which represents the usual white plastics flow within the sampled 3D stream, as well as a sample consisting of only transparent, non-coloured packaging (PP_p_natural_).

#### Extrusion of (non-)packaging blends and preparation of the mechanical test specimens

The PP fractions were extruded at 240°C and 70 rpm screw speed using a single screw extruder without a degassing unit (EX-18-26-1.5; Extron Engineering Oy, Akaa, Finland) and a screw diameter of 18 mm. The material is filtered in front of the outlet zone with a mesh size of 2 mm and the single outlet has a diameter of 3 mm. After extrusion, a sieved fraction of 4 mm was prepared using a mill (Fritsch Pulverisette 19; FRITSCH GmbH, Idar-Oberstein, Germany). This regranulate was used for injection-moulded tensile (impact) strength specimens using a Haake Mini Lab II equipped with a co-rotating twin screw extruder coupled with a Haake Mini Jet II piston injection moulding unit (Thermo Fisher Scientific, Waltham, MA, United States). Extrusion was conducted at 230°C with a screw speed of 100 rpm. The injection moulding specimens were produced at a mould temperature of 40°C, an injection and holding pressure of 350 bar and injection and post-injection times both equalled 10 seconds. At least eight dog-bone tensile (thickness 2 ± 0.2 mm) and tensile impact test specimens (thickness 1.2 ± 0.06 mm) in accordance with ISO 527-2-5A (2012) and ISO 8256/1A (2004) were produced, respectively. Tensile impact test specimens were notched on both sides (each 2 mm) with a CEAST Notch-Vis tool (Instron, Darmstadt, Germany).

#### Tensile (impact) testing of (non-)packaging blends

A universal testing system, comprising a Zwick 050 frame, 2.5 kN load cell and extensometer (Zwick Roell, Ulm, Germany), was used to perform tensile tests on the prepared specimens at a constant velocity of 20 mm·min^−1^. The elastic modulus ( $E_{t}$ ), stress at yield ( $\sigma_{y})$ and elongation at break ( $\varepsilon_{B}$ ) were calculated across six replicate tests. An CEAST 9050 impact pendulum (Instron, Darmstadt, Germany), equipped with a 2 J hammer and 15 g crosshead mass, was utilised to establish the tensile impact strength $(a_{tN}$ ) of the notched samples across at least eight replicates.

#### Thermal, morphological and rheological quality assessment of (non-)packaging blends

The (non-)packaging blend quality was evaluated using gold sputtered fracture surfaces of tensile impact-tested specimens with a Zeiss EVO 10 (Carl Zeiss Microscopy, Oberkochen, Germany) scanning electron microscope (SEM) at an accelerating voltage of 3 kV. Additionally, samples were analyzed using a Q 2000 differential scanning calorimetry (DSC; TA Instruments, New Castle, DE, United States) after extrusion. A 5 ± 0.5 mg mass of each sample was heated twice within a temperature range from 20°C to 200°C and a heating/cooling rate of 10 K·min^−1^. The melting temperature (*T_m_*) of the second heating run was analysed using the maximum of the peak. In addition, the dynamic shear rheology was tested using frequency sweeps on an MCR 302 rheometer (Anton Paar, Graz, Austria). Therefore, a plate–plate system (1 mm gap size) with 230°C and a heating hood purged with nitrogen were used. Deformation was raised logarithmically from 1% to 2% at a frequency ranging from 628 to 0.01 rad·s^−1^. At least three samples were measured. Moreover, the melt flow rate (MFR) was determined for at least eight replicates of each sample according to ISO 1133-1 (2022), method A, under a load of 2.16 kg at 230°C on the MeltFloW basic (Karg Industrietechnik, Krailling, Germany).

### Recycling potential of polypropylene from mixed wastes in Austria

The collected data were utilised to calculate the PP recycling potential from mixed wastes for the region and extrapolate the findings to Austria as a whole. Four scenarios were analysed: (A) separation only in the catchment area of the MRF, (B) an expansion of all existing MRFs in Austria for PP recovery and (C) the sorting of all mixed MSW, bulky and commercial waste. Each recycling mass flow is calculated according to equation (5) with the input mass of mixed wastes in the MRF ( ${\dot{m}}_{MRF,Input}$ ), the mass recovery rate into the 3D fraction ( $R_{3D}$ ), the content of PP (non-)packaging ( $c_{PP\_\left(non-\right)p}$ ) and colour ( $c_{colour}$ ), the sorting mass recovery rate into PP ( $R_{Sort}$ ) and the calculated $R_{PP}$ from equation (4).

(5) $${\dot{m}}_{PP,Recycl}=\;{\dot{m}}_{MRF,Input}\cdot \;R_{3D}\cdot c_{PP\_\left(non-\right)p}\cdot c_{colour}\cdot R_{Sort}\cdot R_{PP}$$

## Results and discussion

### Composition, mass recovery rate, dirt and foreign material content of rigid (non-)packaging plastic blends from mixed waste

**Figure 2.**
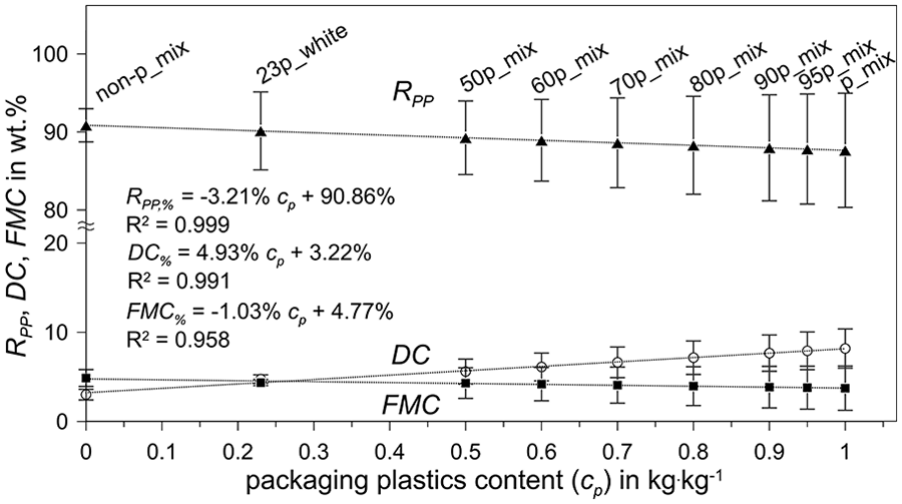
DC (circles), FMC (squares), mass recovery rate ( $R_{PP}$ , triangles) as functions of the packaging plastic content (*c*_p_) of the different PP blends, containing different proportions of packaging (p) and non-packaging (non-p) in natural (transparent, non-coloured), white or mixed colour and the respective linear fits. Values are displayed as mean ± standard deviation. DC: dirt content; FMC: foreign material content; PP: polypropylene.

The DC based on dry matter is generally higher in PP packaging plastics, particularly in transparent, non-coloured variants, with a mean value of 9.7 ± 4.1 wt.%. Thus, a positive linear correlation with a coefficient of determination *R*² of 0.991 is observed between the DC and the packaging plastics content in the sample, as illustrated in Figure 2. The corresponding quantitative values are presented in Table 1; depictions of the sorted fractions can be found in the Supplemental Figure A2(a)–(j). This proportionality is linked to the composition of packaging plastics in mixed MSW. For example, the transparent, non-coloured packaging in the sampled material stream primarily consists of meat packaging containing article inlays, which disintegrate during washing. Moreover, coloured and white packaging, mainly personal hygiene product bottles (shower gel, shampoo, body lotion, etc.), exhibit limited emptiability (Gritsch et al., 2024). These items are also more frequently discarded in mixed MSW rather than source-segregated lightweight packaging waste, likely because consumers perceive them as either unpleasant and unsanitary (Nemat et al., 2022), or the in-house distance from the point of waste generation to the collection point is perceived as too long, for example, in the bathroom (Thoden van Velzen et al., 2019).

**Table 1.** DC based on dry matter, FMC , mass recovery rate ( $R_{PP}$ ) and shares of processing methods within the PP samples sourced from the 3D output of a mixed waste MRF. Values are depicted as means ± standard deviation. Samples are distinguished by type, packaging (index p) or non-packaging (index non-p) and colour (white, coloured and transparent, non-coloured – natural). Processing methods are abbreviated as follows: TF, IM, BM and NI.

Sample type/colour	Mechanical pre-processing			Hand sorting			
	DC (wt.%)	FMC (wt.%)	$R_{PP}$ (wt.%)	TF (wt.%)	IM (wt.%)	BM (wt.%)	NI (wt.%)
PP_p_natural_	9.7 ± 4.1	7.4 ± 6.4	83.1 ± 8.6	70.2	7.1	11.5	7.6^a^
PP_p_white_	9.1 ± 0.6	2.1 ± 0.3	87.6 ± 12.8	33.5	58.4	4.5	3.6
PP_p_coloured_	5.8 ± 1.5	1.2 ± 0.1	92.5 ± 1.5	19.6	47.1	29.9	3.4
PP_non-p_natural_	5.4 ± 0.6	3.9 ± 1.5	81.2 ± 3.4	0	70.9	23.6	5.5
PP_non-p_white_	3.4 ± 0.5	5.0 ± 0.4	90.9 ± 2.6	0	97.6	0.7	1.7
PP_non-p_coloured_	2.2 ± 0.8	4.8 ± 1.5	92.1 ± 1.4	3.5	86.9	7.6	2.0

DC: dirt content; FMC: foreign material content; PP: polypropylene; MRF: material recovery facility; TF: thermoforming; IM: injection moulding; BM: blow moulding; NI: non-identifiable.

a During hand sorting, an additional 3.6 wt.% incorrectly assigned (non-packaging, coloured or white) particles were found in the PPp_natural sample.

The moisture contents of the rigid packaging plastics (11 ± 6.5 wt.%, mean ± standard deviation of a normal distribution, based on wet matter) and rigid non-packaging plastics (1 ± 1 wt.%) have been published by Blasenbauer et al. (2024). Although PP packaging in untreated mixed MSW exhibits an average moisture and DC of around 25 wt.% (Gritsch et al., 2024), this study determined a value for PP_p_mix_ of 19.2 wt.% after sorting in a mixed waste MRF, which is comparable to the separate collection in containers (Gritsch et al., 2024) and much lower in relation to other studies (Thoden van Velzen et al., 2021b). It should be noted that the reported values are influenced by the intensity of mechanical handling applied to the objects, and a certain degree of variability in moisture and DC is to be expected. Moreover, the composition of these dirt adhesions is dissimilar in type and texture.

Sample differences in FMC are mitigated, as packaging materials tend to have more labels and sleeves, whereas non-packaging plastics often contain fillers that increase their intrinsic density above 1000 kg·m^−3^, despite being composed of PP (Möllnitz et al., 2021a; Ragaert et al., 2017). Consequently, the FMC linear fit exhibits a slope ∼1 (cf. Figure 2) with *R*² = 0.958. Mean values for FMC range between 3.7 and 4.9 wt.% for the packaging and non-packaging blends, respectively. Therefore, the mass recovery rate ( $R_{PP})$ is greater for non-packaging material, and a negative linear correlation (*R*² = 0.999) between the packaging plastics content and $R_{PP}$ can be observed. The highest average mass recovery rate of 90.9 wt.% is connected to the non-packaging mix, whereas transparent, non-coloured packaging shows the lowest mean $R_{PP}$ of 83.1 wt.%.

Regarding the processing methods, the results show high injection-moulded contents in non-packaging as well as white and, to a lesser extent, coloured packaging PP, aligning with the range of results from Geier et al. (2024). Transparent, non-coloured packaging exhibits a majority of thermoformed products.

Although the EU places emphasis on increasing the recycling rates of packaging plastics (EU, 2018), there remains substantial neglected potential in non-packaging plastics. Technical indicators suggest that non-packaging plastics recovered from mixed MSW tend to show lower levels of surface soiling and comparatively higher mass recovery rates; however, this does not imply that they are less contaminated overall, especially with regard to legacy additives (Wagner and Schlummer, 2020). Notably, 40 wt.% of plastics in household mixed MSW (Beigl, 2020) and the majority in bulky waste (Merstallinger and Fritz, 2022) are non-packaging plastics, underscoring their potential to advance plastic circularity. This is particularly relevant given that contamination poses challenges in recycling facilities, where wastewater treatment capacity is a limiting factor (Thoden van Velzen et al., 2021b).

### Material properties of rigid (non-)packaging blends from mixed waste

**Figure 3.**
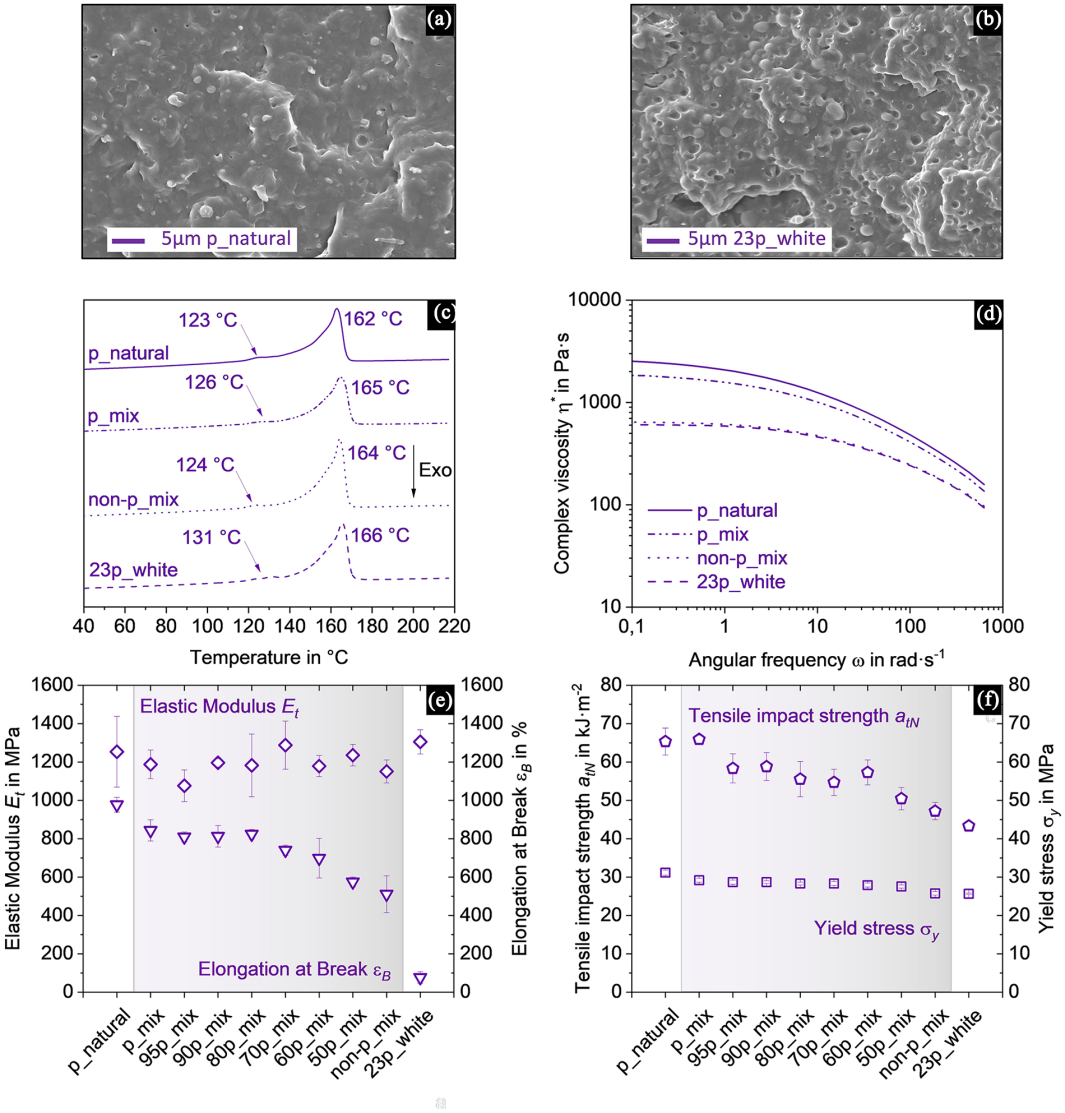
Investigation of PP blends sampled from the 3D output of a mixed waste MRF containing different proportions of packaging (p) and non-packaging (non-p) in natural (transparent, non-coloured), white or mixed colour. SEM images (a) of PP_p_natural_ (b) of PP_23p_white_ verified homogeneity, (c) thermograms of DSC showed PE impurities indicated by arrows. Additional investigated properties are (d) complex viscosity ( $\eta^{*}$ ), (e) elastic modulus ( $E_{t}$ , diamonds) and elongation at break ( $\varepsilon_{B}$ , triangles), (f) tensile impact strength ( $a_{tN}$ , pentagons) and yield stress ( $\sigma_{y}$ , squares). Values are depicted as means ± standard deviation. PP: polypropylene; MRF: material recovery facility; SEM: scanning electron microscope; DSC: differential scanning calorimetry; PE: polyethene.

Considering that the investigated blends result from a waste stream where different molar masses, fillers, additives and non-intentionally added distinct polymers may be combined, the results are as expected, with recyclate from packaging and non-packaging PP showing high overall comparability, as seen in Figure 3(a)–(f). Colour aspects of the regranulate can be seen in the Supplemental Figure A3(a)–(d).

SEM images verified homogeneous mixing quality (Figure 3(a) and (b)). Traces of PE are common in PP recyclate (Alvarado Chacon et al., 2020), so it is not surprising that PE is found in all fractions in a range from 123°C to 131°C by means of a DSC, as indicated by the arrows in Figure 3(c). However, the respective peak between 162°C and 166°C is clearly attributable to PP.

As observable in Figure 3(e), the $E_{t}$ is located between 1076 and 1305 MPa. Commonly, the $E_{t}$ of PP ranges between 1140 and 1550 MPa (Selke et al., 2021). However, for recycled PP, it is usually lower, between 1000 and 1300 MPa typically (Bichler et al., 2024; Domininghaus et al., 2008). The PP_p_mix_ and PP_non-p_mix_ fractions have an $E_{t}$ of 1188 ± 75 and 1151 ± 60 MPa, respectively. Even though the resulting blends demonstrate slightly higher and lower elastic moduli, they are still in the same range.

The highest $\varepsilon_{B}$ can be found for PP_p_natural_ (Figure 3(e)), which also contains the greatest percentage of thermoformed products (Table 1). Furthermore, this aligns with the complex viscosity ( $\eta^{*}$ ; Figure 3(d)), which is also the highest among all the fractions analysed. Generally, a higher molar mass (high viscosity) is expected to result in a higher $\varepsilon_{B}$ (Maier and Schiller, 2016). The blends of PP_p_mix_ remain stable at around 800% until PP_80p_mix_ and consequently decrease continuously for the following blends. It remains unclear why PP_non-p_mix_ still exhibits an $\varepsilon_{B}$ of around 500%. Due to the results of the hand sorting by processing method and the $\eta^{*}$ displayed in Figure 3(d), one could have assumed a similar $\varepsilon_{B}$ of PP_non-p_mix_ compared to PP_23p_white_. Although the processing method can give an indication of the molar mass and thus the elongation at break, it has been shown that there are outliers and, moreover, that injection-moulded parts with an $\varepsilon_{B}$ of 900% also exist (Schlossnikl et al., 2025). Additionally, it is unclear what kind of additives are utilised in the plastics within the waste stream. A variety of such additives or stabilisers are introduced to alter the polymer properties (Maier and Calafut, 1998). Besides, the presence of copolymers could also lead to higher elongations at break (Shao et al., 2023).

The $a_{tN}$ (Figure 3(f)) of the fractions decrease continuously from PP_p_natural_ to PP_23p_white_. With 65.9 ± 1.1 kJ·m^−2^, PP_p_mix_ has the highest impact strength, whereas PP_23p_white_ is characterised by the lowest at 43.3 ± 1.3 kJ·m^−2^. Nonetheless, variations in $a_{tN}$ of PP are quite common (Seier et al., 2023). The $\sigma_{y}$ (Figure 3(f)) remains relatively stable, ranging from 28.7 ± 0.5 MPa of PP_95p_mix_ to 27.6 ± 0.4 MPa of PP_50p_mix_, which is in line with PP recyclate (Domininghaus et al., 2008).

In Table 2, the dynamic rheological data are compared to the MFR. In relation to PP_p_natural_, the molar mass (MM) decreases in all blends and compositions, which is consistent with the complex viscosity and can also be observed from the steadily increasing MFR values, which are indirectly proportional to each other (Baur et al., 2019). Especially for the blends composed of PP_p_mix_ and PP_non-p_mix_, the increasing values of $\omega_{c}$ (decreasing MM) towards the component containing more non-packaging is unambiguous.

**Table 2.** MFR ± standard deviation of rigid PP blends with different amounts of packaging (index p) and non-packaging (index non-p) from mixed waste origin, and results of crossover points at G′ = G″ obtained from dynamic shear rheology, diagrams are displayed in the Supplemental Figures A4–A6.

Sample type/colour	MFR (230°C, 2.16 kg)	Crossover frequency	Crossover modulus	Interpretation with relation to
	MFR (g∙10 min^−1^)	$\omega_{c}$ (rad∙s^−1^)	$G_{c}$ (kPa)	PP_p_natural_
PP_p_natural_	5.6 ± 0.1	78.4 ± 6.6	31.2 ± 0.4	
PP_p_mix_	8.4 ± 0.1	98.0 ± 8.5	29.1 ± 0.9	MM ↓ MMD ↑
PP_95p_mix_	9.8 ± 0.4	105 ± 3.5	29.1 ± 0.7	MM ↓ MMD ↑
PP_90p_mix_	9.5 ± 0.2	117 ± 3.5	28.8 ± 0.5	MM ↓ MMD ↑
PP_80p_mix_	9.6 ± 0.1	122 ± 11.2	29.2 ± 0.6	MM ↓ MMD ↑
PP_70p_mix_	11.0 ± 0.1	125 ± 6.1	29.3 ± 0.7	MM ↓ MMD ↑
PP_60p_mix_	11.7 ± 0.2	144 ± 10.4	29.6 ± 0.7	MM ↓ MMD ↑
PP_50p_mix_	12.9 ± 0.3	157 ± 12.1	30.3 ± 0.3	MM ↓ MMD ↑
PP_non-p_mix_	21.5 ± 0.6	303 ± 29.3	33.0 ± 1.4	MM ↓ MMD ↓
PP_23p_white_	22.7 ± 1.6	284 ± 27.2	31.2 ± 1.5	MM ↓ MMD ∼

MFR: melt flow rate; MM: molar mass; MMD: molar mass distribution; PP: polypropylene.

Non-packaging PP tends to exhibit higher MFRs, which relate to the higher contents of injection-moulded objects (see Table 1) that typically display MFRs between 5 and 100 g·10 min^−1^ (Eriksen et al., 2019). The wider molar mass distribution of the blends can be explained by the blending of two polymers with different MM (Mezger, 2011). However, it is important to note that recycling PP with different grades affects the quality of the recyclate (Geier et al., 2024; Schlossnikl et al., 2025; Thoden van Velzen et al., 2021a; Traxler et al., 2022), which can already be roughly derived by the MFR. Packaging PP exhibits greater variability across processing methods but generally lower MFRs, as Traxler et al. (2022) have shown for yoghurt cups. Non-packaging PP primarily relies on injection moulding. A current challenge lies in the mixed processing methods of PP within PP waste streams and, therefore, mixed grades, making it difficult to achieve uniform recyclates (Geier et al., 2024). However, this mixture also means that the displayed mechanical properties are comparable to the recyclates already available on the market. And since the products for which recyclates are currently used do not have high odour and colour requirements (Christiani and Beckamp, 2020), it is assumed that the use case can meet them.

### Recycling potential of polypropylene from mixed waste in Austria

**Figure 4.**
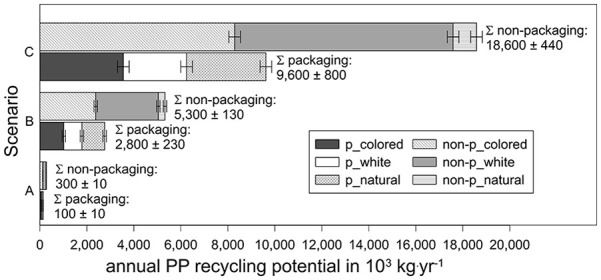
Rigid PP recycling potential from mixed waste in Austria (mean values ± standard deviation) for packaging (p) and non-packaging (non-p) and colour. Depiction of three scenarios: (A) the investigated MRF only, (B) PP-recovery in all mixed waste MRFs in Austria and (C) treatment of all Austrian mixed MSW, bulky and commercial waste in MRFs. PP: polypropylene; MRF: material recovery facility; MSW: municipal solid waste.

The calculated recycling potential of PP is consistently higher for non-packaging waste across all scenarios, as illustrated in Figure 4. Packaging PP demonstrates considerable recycling potential, particularly for transparent, non-coloured packaging, whereas non-packaging PP shows greater potential for coloured items. If the entire volume of mixed MSW, bulky waste and commercial waste were directed to a mixed waste MRF, calculated in scenario (C), approximately 2.8 × 10^7^ kg·yr⁻¹ of PP could be recycled. This would translate to around 7 wt.% of plastic in mixed waste streams being diverted from incineration, but would necessitate the construction of new MRFs as well as the upgrading of existing ones.

Coloured PP offers the greatest recovery potential, and the colour of post-consumer plastic severely influences its recyclability and recyclate application (Gritsch et al., 2024; RecyClass, 2025; Shamsuyeva and Endres, 2021). Largely, transparent and white fractions are preferred, as they allow for broader applications, whereas coloured materials often result in visually inconsistent recyclates with limited marketability (Faraca and Astrup, 2019). Colouring is generally irreversible, and removal is technically challenging and cost-intensive (Shamsuyeva and Endres, 2021). Additionally, certain pigments may contain substances of concern (SOCs; Faraca and Astrup, 2019). Although this study differentiated coloured, white, and transparent, non-coloured PP, no quantitative colourimetric analysis was conducted, representing a methodological limitation. Nevertheless, given the material’s origin from mixed MSW, it is inherently unsuitable for high-value or food-contact applications due to contamination and odour issues, as further discussed in section ‘Beyond mechanical properties: Contamination considerations’.

An additional factor to consider is that while recyclates can be produced from recovered (non-)packaging plastics, their competitiveness is severely undermined by the low cost of imported virgin material (Larrain et al., 2021). Thus, increasing plastic recycling within Europe remains challenging, with companies at possible risk of insolvency. Nevertheless, the minimum recycled contents for packaging stipulated in the EU packaging waste regulation are expected to drive the demand for recycled plastics (EU, 2024).

Alternatively, if all existing MRFs in Austria were upgraded for PP recovery without constructing additional facilities in scenario (B) (Lipp and Lederer, 2025), approximately 8.1 × 10^6^ kg·yr⁻¹ of PP could be recycled, equivalent to 2 wt.% of plastic in mixed waste streams.

Generally, reducing the amount of plastic in mixed MSW and incineration through improved separate collection and subsequent recycling has been found to have environmental as well as economic advantages by reducing CO_2_ emissions and increasing throughputs in incineration plants due to the removal of high calorific plastics (Kranzinger et al., 2017). Furthermore, Feil et al. (2017) state that expanding mechanical biological treatment plant capacities to around 5.0 × 10^7^ kg·yr^−1^ can lead to an economically feasible recovery of plastics from mixed wastes.

On the contrary, recovering plastics from mixed wastes is a more energy-intensive process, and the environmental savings are sensitive to the energy mix utilised (Bernstad et al., 2011; Jiang and Bateer, 2025; Nordahl and Scown, 2024). Moreover, the use of plastic waste as refuse-derived fuel to substitute fossil fuels in energy-intensive industries must be considered (Cimpan and Wenzel, 2013). That said, the impact of removing 2–7 wt.% of plastics from mixed wastes can be regarded as relatively minor. Overall, an optimal balance for recycling is likely to fall somewhere in between (Ragossnig and Schneider, 2017).

### Beyond mechanical properties: Contamination considerations

One of the biggest barriers to the widespread use of recycled plastics is the presence of contaminants, particularly odorous volatiles and SOCs (Cabanes et al., 2020; De Somer et al., 2022; Eriksen et al., 2018; García Ibarra et al., 2018; Horodytska et al., 2020; Roosen et al., 2022; Strangl et al., 2018, 2020, 2021). This challenge applies to all post-consumer plastics but is especially prevalent in recyclates derived from commingled collections or mixed MSW due to cross-contamination (Cabanes et al., 2020; Roosen et al., 2023). Thereby, contaminants originate from a variety of sources, including organic matter (Cabanes et al., 2020), fragrances from cleaning products and personal hygiene (Horodytska et al., 2020), waste from electrical and electronic equipment (WEEE) containing additives such as brominated flame retardants, and consumer misuse of packaging (Geueke et al., 2018). Polyolefins and their relatively high permeability present a specific challenge (De Somer et al., 2022; Geueke et al., 2018). Compared to polyesters, PP exhibits significantly higher diffusion coefficients, resulting in a greater tendency for chemical migration (Dole et al., 2006; Palkopoulou et al., 2016; Zhang et al., 2025). Generally, a range of plastic additives, such as certain phthalates, lead and cadmium compounds, can be considered SOCs; however, their use is predominantly associated with polyvinyl chloride (Hahladakis et al., 2018; Wagner and Schlummer, 2020).

Regarding odour, various strategies have been explored to mitigate contamination in polyolefin recycling: The EREMA ReFresher system uses hot air treatment to remove volatile compounds from extruded pellets (EREMA, 2024; Roosen et al., 2022). Similarly, hot air devolatilisation conducted before extrusion has been shown to effectively reduce odour without compromising mechanical properties (Bichler et al., 2024). Pre-extrusion methods such as hot washing, the use of organic solvents, or specialised detergents also contribute to odour reduction (De Somer et al., 2022; Roosen et al., 2022; Strangl et al., 2021) as well as approaches such as probiotic bacteria pre-treatment prior to washing (Lok et al., 2020).

In the present study, the odour was not assessed, as the focus was on mechanical characterisation to demonstrate that PP recycled from mixed waste streams has no inherent mechanical limitations compared to source-segregated PP. Nonetheless, odour remains a critical issue, and research specific to mixed MSW recyclates is still limited (Cabanes et al., 2020; Maaskant-Reilink et al., 2020). Further investigation is warranted, particularly as higher contamination levels in mixed waste call for improved washing processes (Cabanes et al., 2020). Here, maintaining washing media quality, which plays a key role in decontamination efficiency (De Somer et al., 2022), is crucial. In the context of more heavily contaminated mixed MSW, this raises important questions, such as the cost-efficiency of washing systems, strategies to control microbial growth, as well as water treatment and recirculation techniques (De Somer et al., 2022; Strangl et al., 2020).

In addition, SOCs, especially those with migration potential, pose a notable risk to consumer health and safety regarding recycled plastics (García Ibarra et al., 2018; Horodytska et al., 2020). The recycled PP from mixed MSW analysed in this study cannot be used for food contact or other critical applications due to the current legal requirements. It should be considered a complementary stream to source-segregated recycling, acknowledging that full consumer compliance with waste separation is unlikely to be achieved, especially in urban environments (Feil et al., 2017; Haupt et al., 2018; Lederer et al., 2022; Schuch et al., 2023).

SOCs are often associated with specific product types, like WEEE or engine oil packaging, which are more prevalent in mixed MSW. Emerging sensor-based waste sorting technologies, including artificial intelligence-assisted object recognition for specific products, offer promising ways to reduce contamination (Ihsanullah et al., 2022). Manual sorting remains essential even for separately collected waste (Aberger et al., 2025) and can further help reduce the presence of SOC-containing items in streams derived from mixed MSW. Additionally, logistical measures in MRFs, such as input-dependent recovery, that is, choosing not to recover material from particular input waste origins, can further support recyclate quality.

As demand for recycled plastics grows, a hierarchical approach to their application is needed: separately collected materials for high-quality recycling and mixed MSW-derived recyclates for lower-grade applications. Given the presence of legacy additives, current EU policy generally requires the same limits for hazardous substances in both virgin and recycled materials, with exceptions only for clearly defined, low-risk uses (European Commission, 2020; Lahl and Zeschmar-Lahl, 2024). Implementing quality management systems could improve safety and consistency for plastics recycling from mixed MSW; their form and scope should be the subject of further research.

## Conclusion

The quality, regarding mechanical properties, of recycled PP produced from mixed waste and separate collection are essentially comparable, which underscores the capability of utilising the recovery and recycling of plastics from mixed MSW. This article investigated the recycling and mechanical pre-processing of PP from such mixed waste origin, focusing on rigid packaging and non-packaging plastics. To achieve this, output streams from a mixed waste MRF that treats mixed MSW, bulky and commercial waste were sampled, and the contained PP was manually sorted out. The material underwent washing and sink-float separation to determine dirt and FMC, followed by extrusion, tensile testing and quality assessment of the blends using thermal, morphological and rheological analyses.

On average, the PP packaging was found to be no more soiled than that obtained through separate collection, although the dirt differs in composition and its level of hazard. Moreover, it was confirmed that different PP blends originating from a mixed waste MRF result in a recyclate with comparable mechanical properties to those already on the market. These results counter the frequent rejection of plastics recycling from mixed MSW due to concerns over non-packaging and its interplay with mechanical properties. Generally, the findings highlight that these challenges are often overstated. They underscore the potential for recycling in urban centres with low separate collection capture rates (Feil et al., 2017), which would further reduce incineration and primary resource dependence. Nevertheless, contamination remains a defining challenge for recycling, and although this study has shown that mechanical qualities can be met, odour and chemical safety must be rigorously addressed. The findings demonstrate that recycling PP from mixed wastes can potentially be a viable option once SOCs and the influence of contaminants are considered.

Future research should address several key areas. Although evaluation of the mechanical properties is partially possible if information on the composition of the processing method is available, it is not definitive due to the inherent variability in waste streams, as seen with blends PP_23p_white_ and PP_non-p_mix_. This issue is exacerbated by the fact that *Design for Recycling* is still in its infancy. Nonetheless, all fractions display acceptable mechanical properties for non-critical injection moulding applications, with PP_p_natural_ demonstrating particularly favourable characteristics, making it suitable for potential thermoforming applications.

However, the economic feasibility as well as energy and environmental implications of recovering plastics from mixed waste remain uncertain and need further investigation, albeit lessons can be drawn from the practice in other EU countries. Furthermore, the presence of SOCs (Hahladakis et al., 2018; Sormunen et al., 2022) should be systematically researched to ensure the safety and quality of recyclates. This aspect is vital regarding the long-term applicability of recycled materials and cascading recycling loops. These challenges should be met with coordinated efforts across the product design, sorting, washing and policy domains. If the identified barriers are addressed, recycling plastics from mixed waste origin can contribute to sustainable waste management and circularity goals.

## Supplemental Material

sj-pdf-1-wmr-10.1177_0734242X251357137 – Supplemental material for Recycling rigid polypropylene from mixed waste: Does the origin affect mechanical recyclate quality? Supplemental material, sj-pdf-1-wmr-10.1177_0734242X251357137 for Recycling rigid polypropylene from mixed waste: Does the origin affect mechanical recyclate quality? by Anna-Maria Lipp, Jessica Schlossnikl, Isabelle Gentgen, Thomas Koch, Vasiliki‑Maria Archodoulaki and Jakob Lederer in Waste Management & Research
