# Supplementary material for: Recycling rigid polypropylene from mixed waste: Does the origin affect mechanical recyclate quality?
Source: Waste Manag Res. 2025 Aug 20;44(1):88–100. doi: 10.1177/0734242X251357137 (PMC12728084; doi:10.1177/0734242X251357137)
Supplement: sj-pdf-1-wmr-10.1177_0734242X251357137 – Supplemental material for Recycling rigid polypropylene from mixed waste: Does the origin affect mechanical recyclate quality? [file sj-pdf-1-wmr-10.1177_0734242X251357137.pdf]

Supplementary Material

Recycling Rigid Polypropylene from Mixed Waste: Does the Origin Affect Mechanical Recyclate Quality?

Anna-Maria Lipp[1,*], Jessica Schlossnikl[2], Isabelle Gentgen[2,3], Thomas Koch[2], Vasiliki-Maria Archodoulaki[2], Jakob Lederer[1]

[1] Christian Doppler Laboratory for a recycling-based Circular Economy, Institute of Chemical, Environmental and Bioscience Engineering, TU Wien, Getreidemarkt 9, 1060 Wien, Austria

[2] Institute of Materials Science and Technology, Faculty of Mechanical and Industrial Engineering, TU Wien, Gumpendorferstrasse 7, Object 8, 1060 Vienna, Austria

[3] Institute for Microtechnology and Photonics, Hochschule OST (Eastern Switzerland University of Applied Sciences), Werdenbergstrasse 4, 9471 Buchs SG, Switzerland

*Correspondence: anna-maria.lipp@tuwien.ac.at

List of Figures

Figure A 1. Different packaging and non-packaging fractions when sorted into different processing methods. 5

Figure A 2. Different packaging and non-packaging fractions before the mechanical pre-processing and after washing. 7

Figure A 3. Regranulate of the different PP fractions. 8

Figure A 4. Storage (G´, dashed) and loss (G´´, solid) modulus of PPp_natural and PP23p_white. 10

Figure A 5. Storage (G´, dashed) and loss (G´´, solid) modulus of PPp_mix and corresponding blends with 95-80wt.% PPp_mix. 10

Figure A 6. Storage (G´, dashed) and loss (G´´, solid) modulus of blends with 70-50wt.% PPp_mix and component PPnon-p_mix. 11

List of Tables

Table A 1. Dirt content (DC) based on dry matter, foreign material content (FMC), mass recovery rate (RPP) and shares of processing methods within the polypropylene (PP) samples sourced from the 3D output of a mixed waste material recovery facility (MRF) of primary (above) and secondary results (calculated, below). Values are depicted as means ± standard deviation. Samples are distinguished by type, packaging (index p) or non-packaging (index non-p), and colour (white, coloured and transparent non-coloured - natural). Processing methods are abbreviated the following: thermoforming (TF), injection moulding (IM), blow moulding (BM), non-identifiable (NI). Values are displayed as mean ± standard deviation. 9

Table A 2. Data on mechanical properties of recycled PP. Values are displayed as mean ± standard deviation. 11

Table A 3. Data for PP recycling potential calculation. Mass recovery rates (R) and concentrations (c) are displayed as fractions in $t \cdot t^{-1}$. References: [1] Blasenbauer et al. (2024), [2] Lipp and Lederer (2025), [3] BMK (2024)........ 12

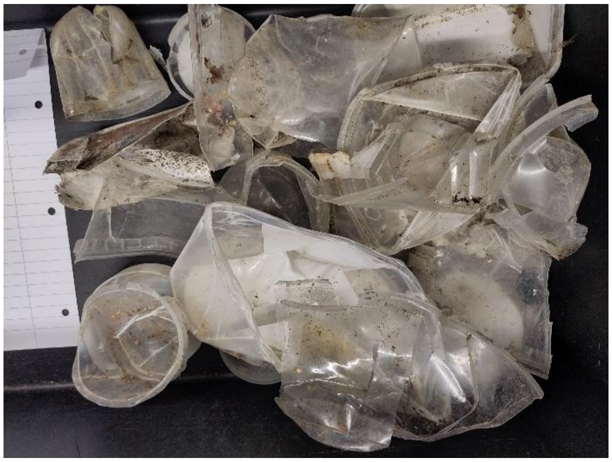

a) natural PP packaging injection-moulded

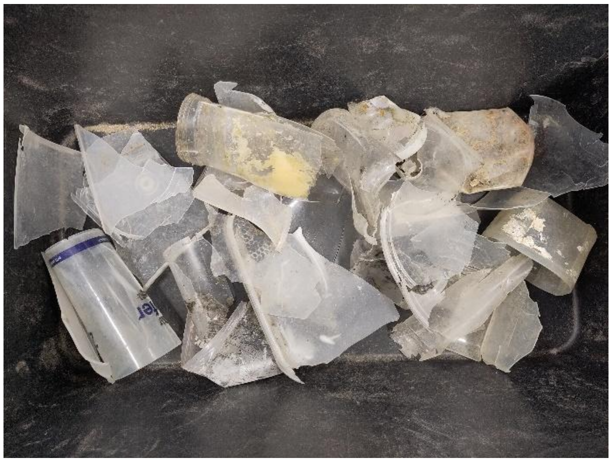

b) natural PP non-packaging injection-moulded

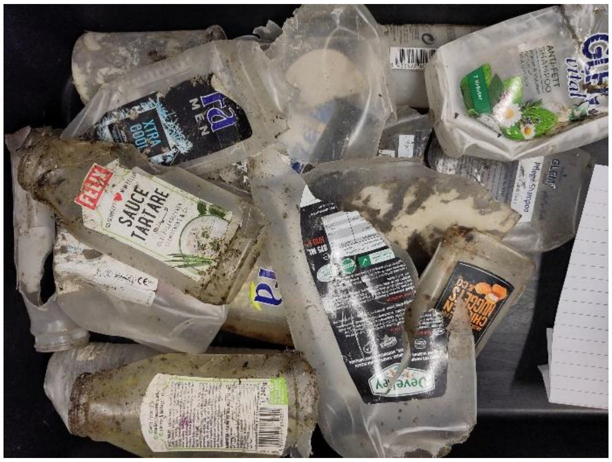

c) natural PP packaging blow-moulded

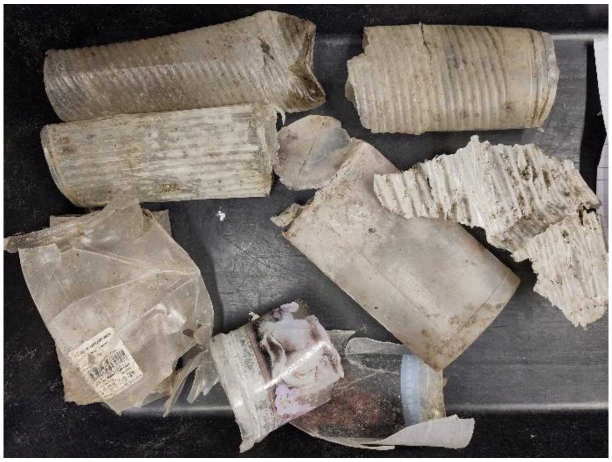

d) natural PP non-packaging blow-moulded

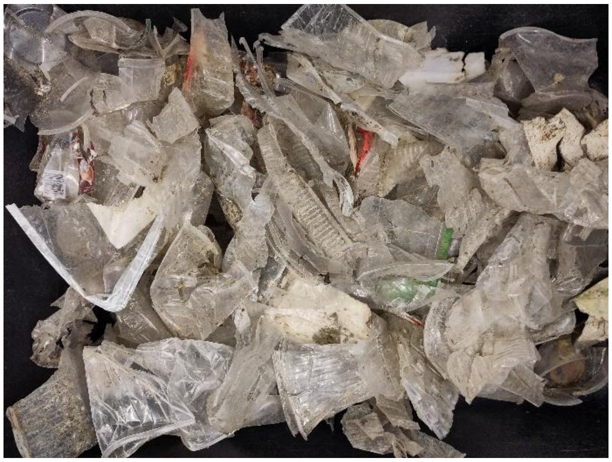

e) natural PP packaging thermoformed

f) natural PP non-packaging thermoformed was nonexistent

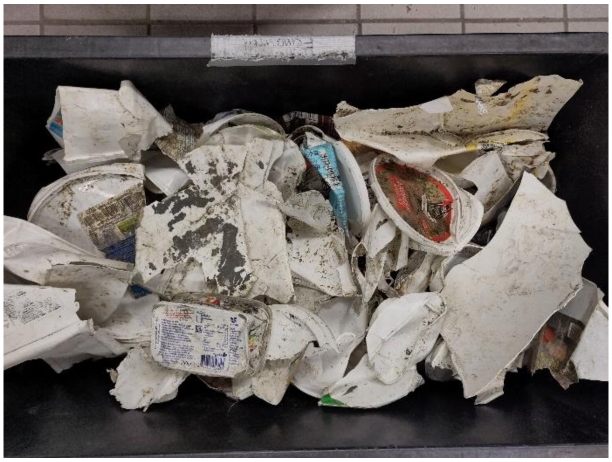

g) White PP packaging injection-moulded

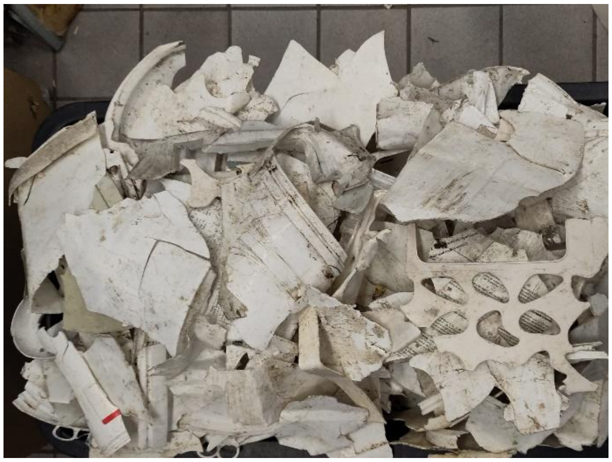

h) White PP non-packaging injection moulded

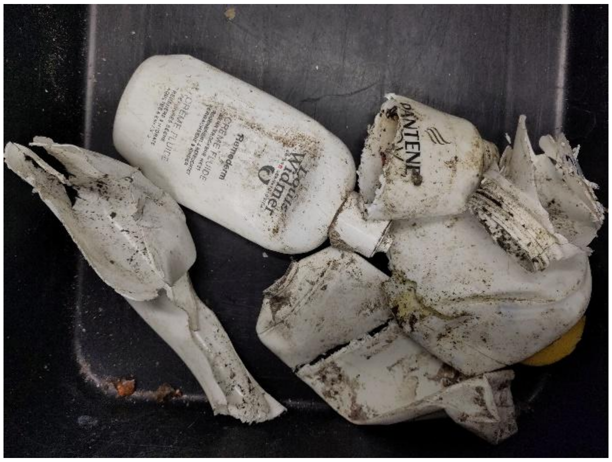

i) White PP packaging blow-moulded

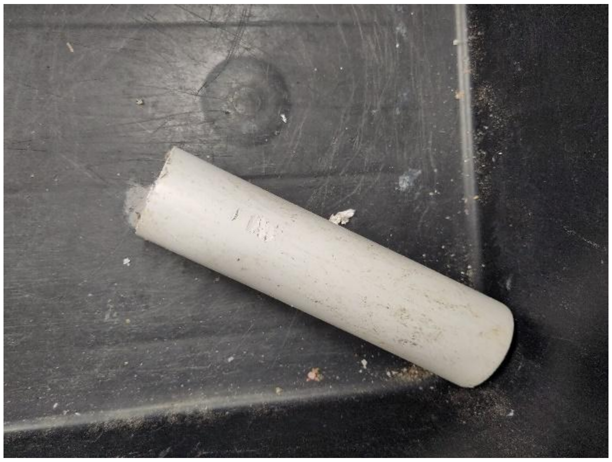

j) White PP non-packaging blow-moulded

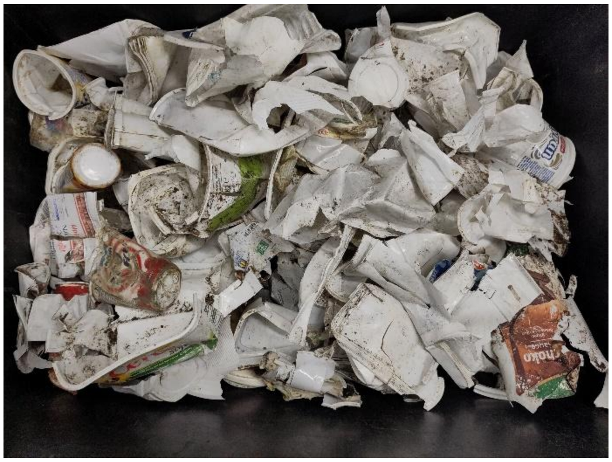

k) White PP packaging thermoformed

l) White PP non-packaging thermoformed was nonexistent

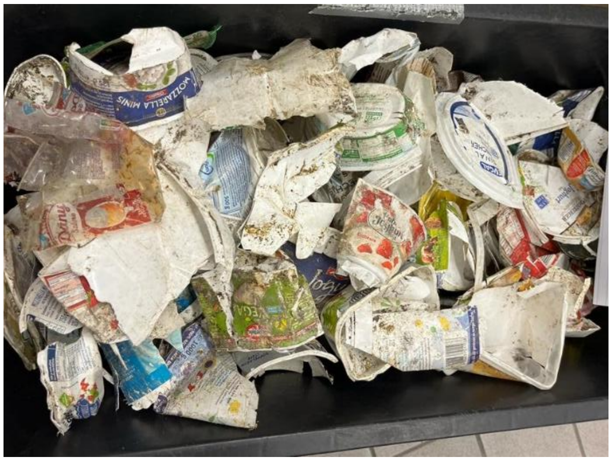

m) coloured PP packaging injection-moulded

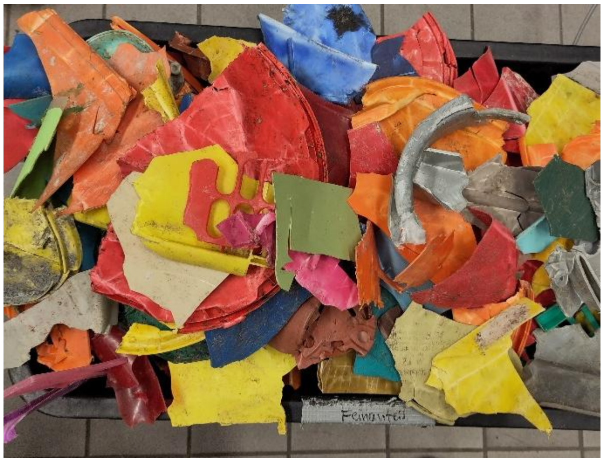

n) coloured PP non-packaging injection-moulded

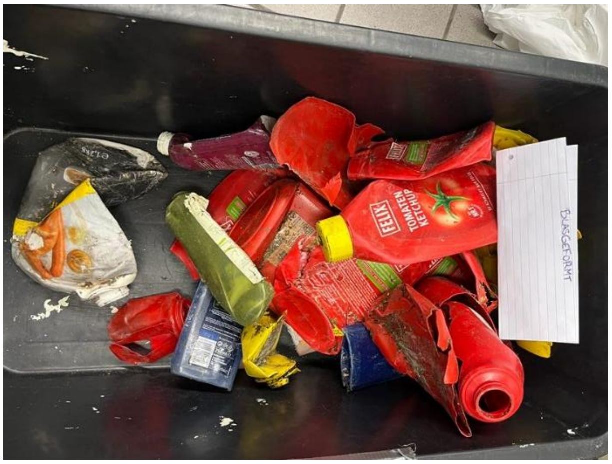

o) coloured PP packaging blow-moulded

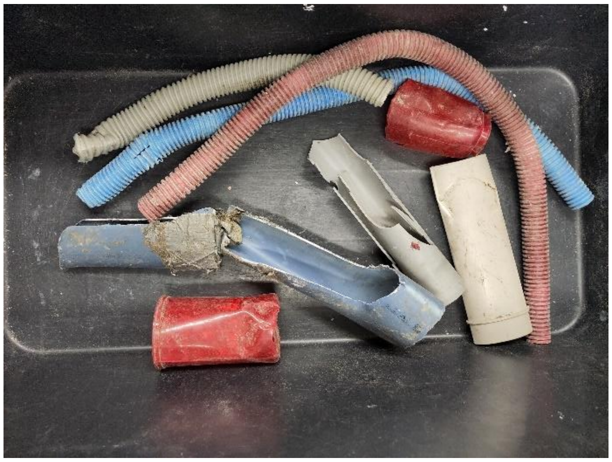

p) coloured PP non-packaging blow-moulded

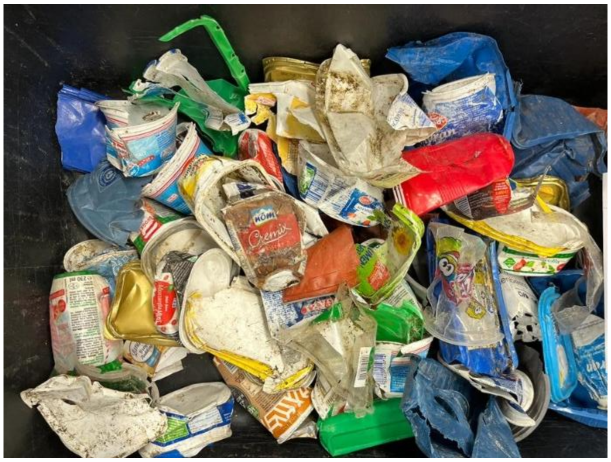

q) coloured PP packaging thermoformed

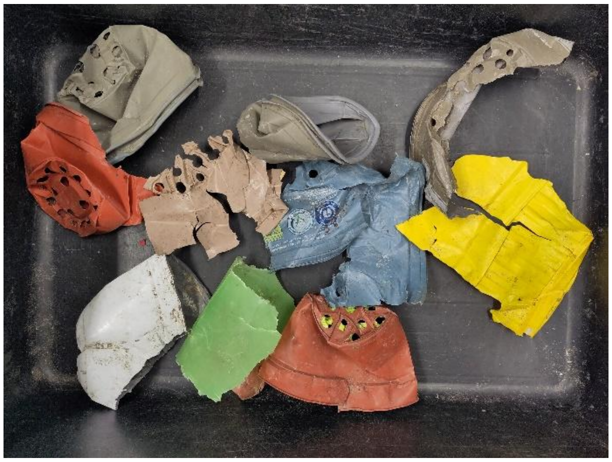

r) coloured PP non-packaging thermoformed

Figure A 1. Different packaging and non-packaging fractions when sorted into different processing methods.

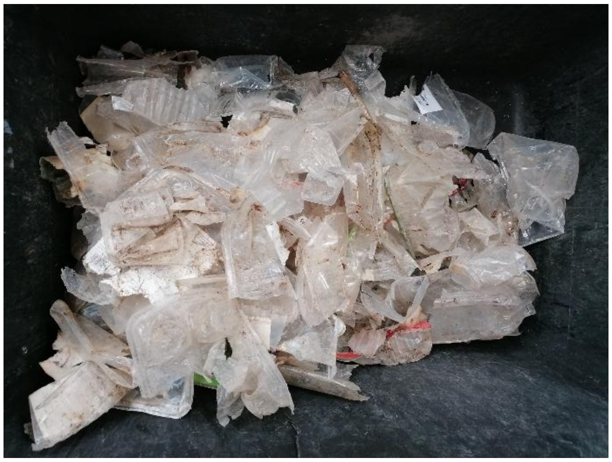

a) natural PP packaging

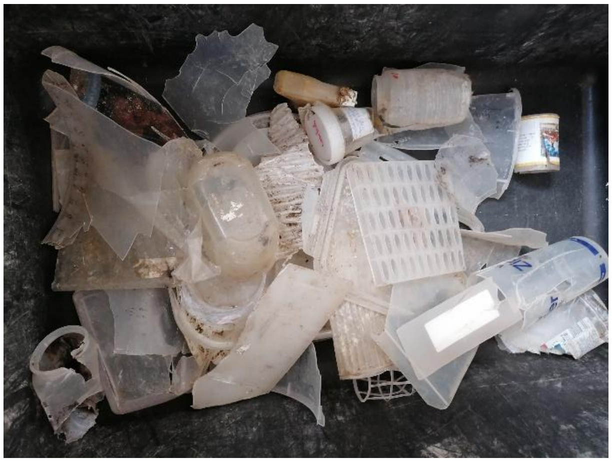

b) natural PP non-packaging

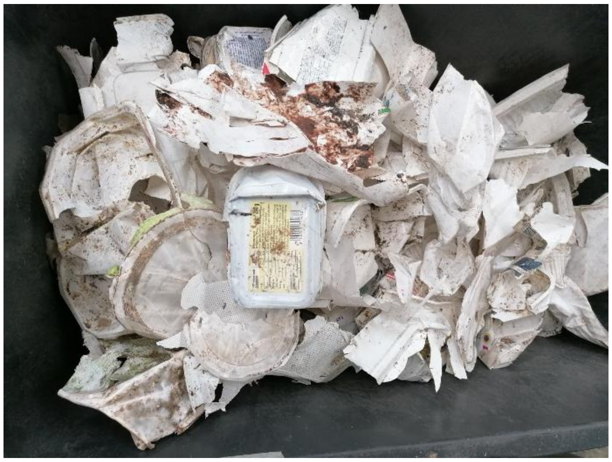

c) white PP packaging

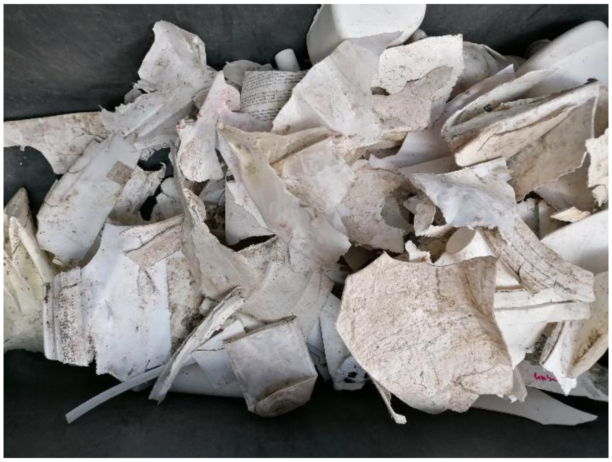

d) white PP non-packaging

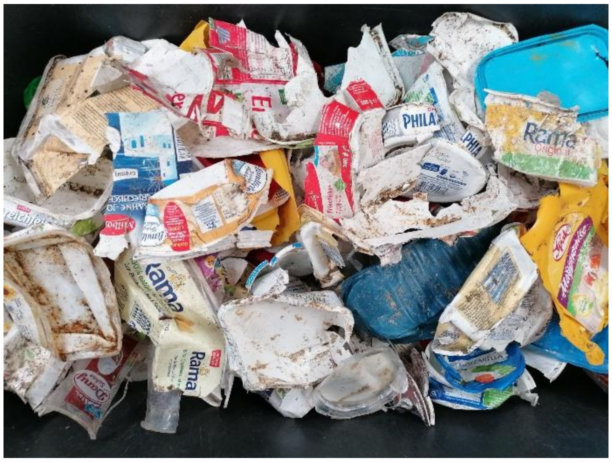

e) coloured PP packaging

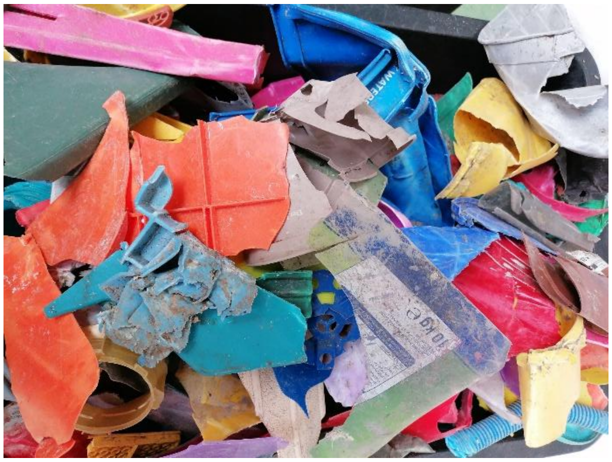

f) coloured PP non-packaging

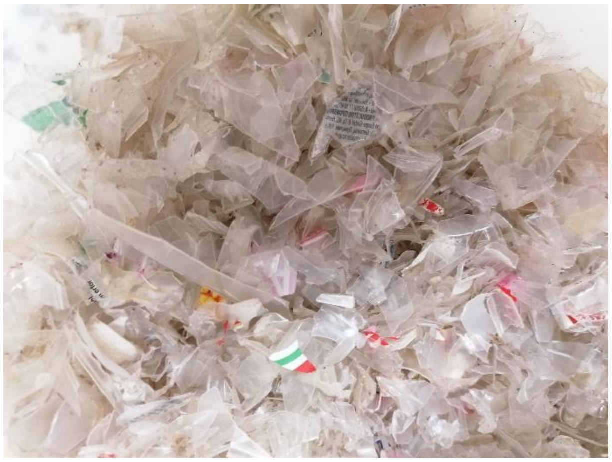
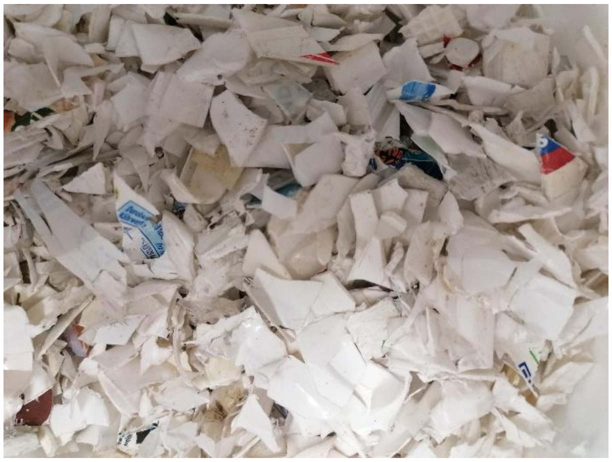

g) $PP_{p\_natural}$ after washing

h) PP_{23p_white} after washing

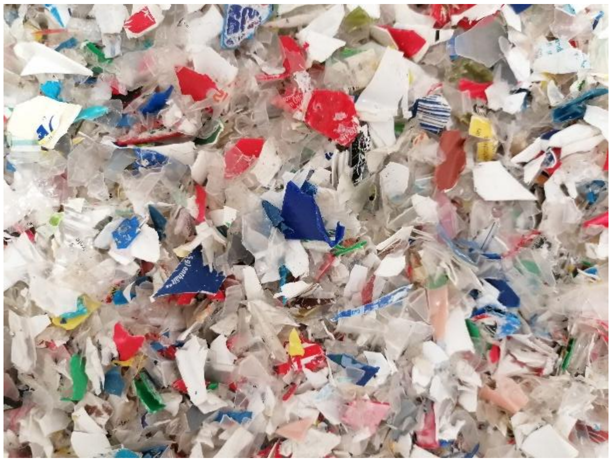
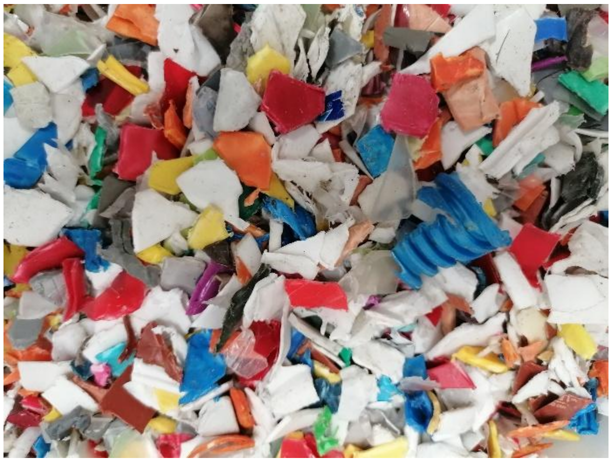

i) PP_{p_mix} after washing

j) PP_{non-p_mix} after washing

Figure A 2. Different packaging and non-packaging fractions before the mechanical pre-processing and after washing.

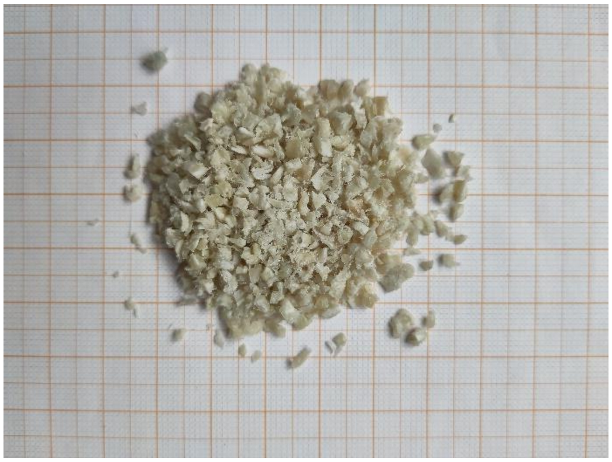

a) $PP_{p\_natural}$

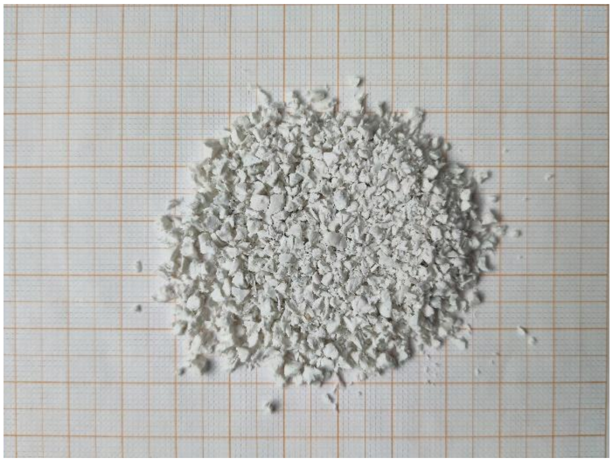

b) PP_{23p_white}

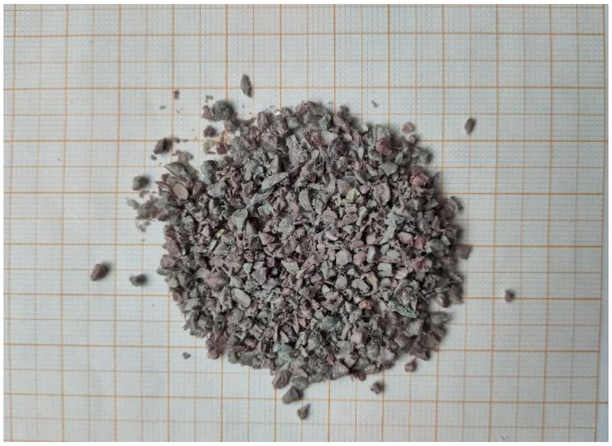

c) PP_{p_mix}

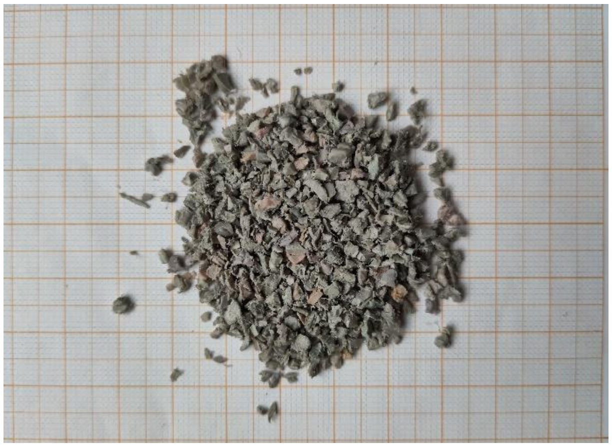

d) $PP_{non\text{-}p\_mix}$

Figure A 3. Regranulate of the different PP fractions.

Table A 1. Dirt content (DC) based on dry matter, foreign material content (FMC), mass recovery rate (R_{PP}) and shares of processing methods within the polypropylene (PP) samples sourced from the 3D output of a mixed waste material recovery facility (MRF) of primary (above) and secondary results (calculated, below). Values are depicted as means ± standard deviation. Samples are distinguished by type, packaging (index p) or non-packaging (index non-p), and colour (white, coloured and transparent non-coloured - natural). Processing methods are abbreviated as follows: thermoforming (TF), injection moulding (IM), blow moulding (BM), non-identifiable (NI). Values are displayed as mean ± standard deviation.

		Mechanical Pre-Processing			Hand Sorting			
Sample Type/Colour	Description	DC in wt.%	FMC in wt.%	R_{PP} in wt.%	TF in wt.%	IM in wt.%	BM in wt.%	NI in wt.%
$PP_{p\_natural}$	manually sorted out from sampled MRF 3D Material stream	9.7±4.1	7.4±6.4	83.1±8.6	70.2	7.1	11.5	7.6*
PP_{p_white}		9.1±0.6	2.1±0.3	87.6±12.8	33.5	58.4	4.5	3.6
$PP_{p\_coloured}$		5.8±1.5	1.2±0.1	92.5±1.5	19.6	47	29.9	3.4
$PP_{non\text{-}p\_natural}$		5.4±0.6	3.9±1.5	81.2±3.4	0	70.9	23.6	5.5
$PP_{non\text{-}p\_white}$		3.4±0.5	5.0±0.4	90.9±2.6	0	97.6	0.7	1.7
$PP_{non\text{-}p\_coloured}$		2.2±0.8	4.8±1.5	92.1±1.4	3.5	86.9	7.6	2.0
The aforementioned samples were recombined to produce the samples listed below, which were subjected to extrusion. The DC, FMC, R_{PP} and proportions of the processing method were calculated accordingly.								
PP_{p_mix}	37 wt.% $PP_{p\_natural}$, 28 wt.% PP_{p_white}, 35 wt.% $PP_{p\_coloured}$	8.2±2.2	3.7±2.5	87.6±7.3	42.2	35.4	16.0	5.0
$PP_{non\text{-}p\_mix}$	6 wt.% $PP_{non\text{-}p\_natural}$, 50 wt.% $PP_{non\text{-}p\_white}$, 44 wt.% $PP_{non\text{-}p\_coloured}$	3.0±0.6	4.9±0.9	90.9±2.1	1.5	91.3	5.1	2.1
PP_{23p_white}	23 wt.% PP_{p_white}, 27 wt.% $PP_{non\text{-}p\_white}$	4.7±0.5	4.4±0.3	90.1±5.0	7.7	88.6	1.6	2.1
PP_{95p_mix}	Different combinations of PP_{p_mix} and $PP_{non\text{-}p\_mix}$	7.9±2.1	3.8±2.4	87.8±7.0	40.2	38.2	15.4	4.9
PP_{90p_mix}		7.7±2.0	3.8±2.3	88.0±6.8	38.1	41.0	14.9	4.7
PP_{80p_mix}		7.1±1.9	4.0±2.2	88.3±6.3	34.1	46.6	13.8	4.4
PP_{70p_mix}		6.6±1.7	4.1±2.0	88.6±5.8	30.0	52.2	12.7	4.1
PP_{60p_mix}		6.1±1.6	4.2±1.9	88.9±5.2	25.9	57.8	11.6	3.8
PP_{50p_mix}		5.6±1.4	4.3±1.7	89.3±4.7	21.9	63.4	10.5	3.5

* during hand sorting, an additional 3.63 wt.% incorrectly assigned (non-packaging, coloured, or white) particles were found in the $PP_{p\_natural}$ sample

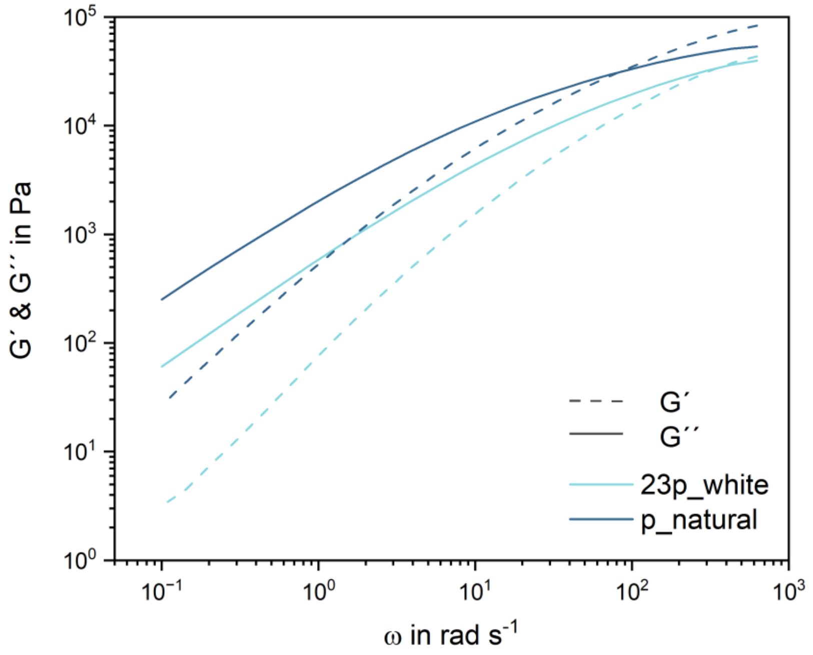

Figure A 4. Storage (G´, dashed) and loss (G´´, solid) modulus of $PP_{p\_natural}$ and PP_{23p_white}.

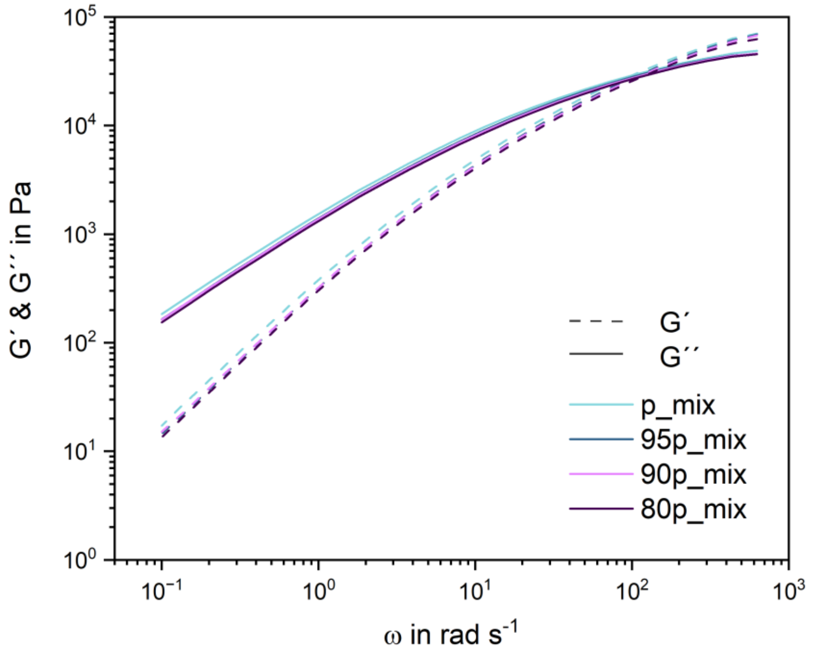

Figure A 5. Storage (G´, dashed) and loss (G´´, solid) modulus of PP_{p_mix} and corresponding blends with 95-80wt.% PP_{p_mix}.

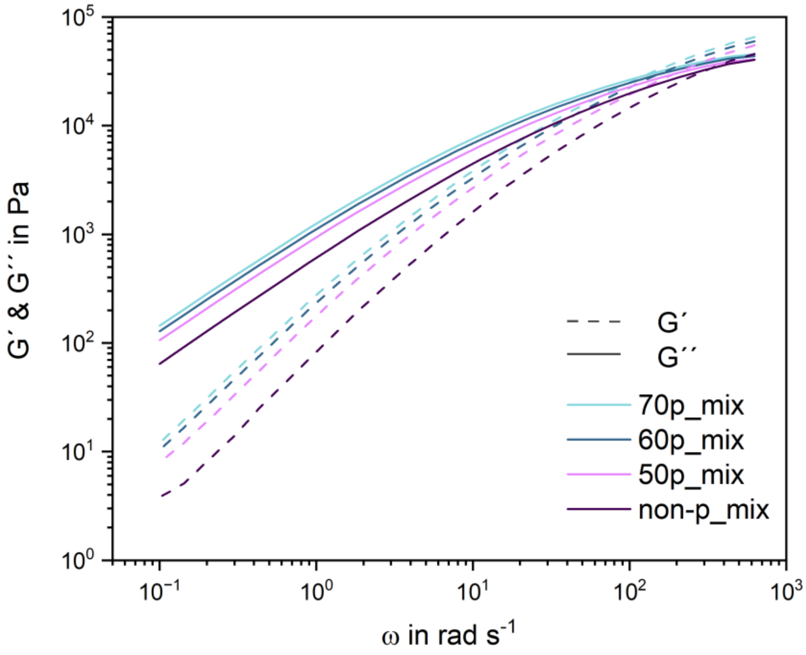

Figure A 6. Storage (G´, dashed) and loss (G´´, solid) modulus of blends with 70-50wt.% PP_{p_mix} and component $PP_{non\text{-}p\_mix}$.

Table A 2. Data on mechanical properties of recycled PP. Values are displayed as mean ± standard deviation.

	Elastic Modulus E_t MPa			Yield stress σ_y MPa			Elongation at break ε_B %			Tensile impact test a_{tN} kJ/m²		
p_natural	1253	±	185	31.2	±	0.7	977	±	38	65.3	±	3.5
p_mix	1188	±	75	29.2	±	0.5	844	±	56	65.9	±	1.1
95p_mix	1076	±	82	28.7	±	0.5	810	±	26	58.3	±	3.8
90p_mix	1196	±	21	28.7	±	0.4	813	±	56	58.8	±	3.6
80p_mix	1182	±	164	28.3	±	0.4	824	±	25	55.5	±	4.6
70p_mix	1288	±	125	28.3	±	0.5	740	±	27	54.7	±	3.4
60p_mix	1179	±	55	27.9	±	0.5	698	±	103	57.3	±	3.3
50p_mix	1236	±	55	27.6	±	0.4	575	±	26	50.5	±	2.9
non-p_mix	1151	±	60	25.8	±	0.5	511	±	96	47.2	±	2.2
23p_white	1305	±	63	25.6	±	0.2	77	±	30	43.3	±	1.3

Table A 3. Data for PP recycling potential calculation. Mass recovery rates (R) and concentrations (c) are displayed as fractions in $t \cdot t^{-1}$. References: [1)] Blasenbauer et al. (2024), [2)] Lipp and Lederer (2025), [3)] BMK (2024).

	Category		$\dot{m}_{MRF,Input}$ in $t \cdot yr^{-1}$	R_{3D}	$c_{PP\_(non-)p}$	c_{color}	R_{Sort}	R_{PP}	$\dot{m}_{PP,Recycl}$ in $t \cdot yr^{-1}$	Sum categories in $t \cdot yr^{-1}$	Sum total in $t \cdot yr^{-1}$
Scenario A	packaging	natural	80000 [1)]	0.12 [1), 3)]	0.022 [1)]	0.37	0.75 [1)]	0.83 ± 0.06	49.98 ± 3.67		
	packaging	white	80000 [1)]	0.12 [1), 3)]	0.022 [1)]	0.28	0.75 [1)]	0.81 ± 0.09	37.00 ± 4.14	136.85 ± 8.40	
	packaging	colored	80000 [1)]	0.12 [1), 3)]	0.022 [1)]	0.35	0.75 [1)]	0.88 ± 0.01	49.88 ± 0.59		416.43 ± 10.96
	non-packaging	natural	80000 [1)]	0.12 [1), 3)]	0.041 [1)]	0.06	0.75 [1)]	0.91 ± 0.00	16.53 ± 0.05		
	non-packaging	white	80000 [1)]	0.12 [1), 3)]	0.041 [1)]	0.5	0.75 [1)]	0.93 ± 0.00	140.19 ± 0.54	279.58 ± 2.56	
	non-packaging	colored	80000 [1)]	0.12 [1), 3)]	0.041 [1)]	0.44	0.75 [1)]	0.92 ± 0.01	122.86 ± 1.97		
Scenario B	packaging	natural	1105000 [2)]	0.17 [1), 3)]	0.022 [1)]	0.37	0.75 [1)]	0.83 ± 0.06	966.42 ± 71.01		
	packaging	white	1105000 [2)]	0.17 [1), 3)]	0.022 [1)]	0.28	0.75 [1)]	0.81 ± 0.09	715.40 ± 79.99	2646.40 ± 162.50	
	packaging	colored	1105000 [2)]	0.17 [1), 3)]	0.022 [1)]	0.35	0.75 [1)]	0.88 ± 0.01	964.57 ± 11.50		8052.68 ± 211.95
	non-packaging	natural	1105000 [2)]	0.17 [1), 3)]	0.041 [1)]	0.06	0.75 [1)]	0.91 ± 0.00	319.57 ± 0.96		
	non-packaging	white	1105000 [2)]	0.17 [1), 3)]	0.041 [1)]	0.5	0.75 [1)]	0.93 ± 0.00	2710.87 ± 10.42	5406.29 ± 49.45	
	non-packaging	colored	1105000 [2)]	0.17 [1), 3)]	0.041 [1)]	0.44	0.75 [1)]	0.92 ± 0.01	2375.84 ± 38.07		
Scenario C	packaging	natural	3937000 [3)]	0.17 [1), 3)]	0.022 [1)]	0.37	0.75 [1)]	0.83 ± 0.06	3372.98 ± 247.85		
	packaging	white	3937000 [3)]	0.17 [1), 3)]	0.022 [1)]	0.28	0.75 [1)]	0.81 ± 0.09	2496.89 ± 279.17	9236.41 ± 567.15	
	packaging	colored	3937000 [3)]	0.17 [1), 3)]	0.022 [1)]	0.35	0.75 [1)]	0.88 ± 0.01	3366.54 ± 40.13		28105.34 ± 739.73
	non-packaging	natural	3937000 [3)]	0.17 [1), 3)]	0.041 [1)]	0.06	0.75 [1)]	0.91 ± 0.00	1115.37 ± 3.35		
	non-packaging	white	3937000 [3)]	0.17 [1), 3)]	0.041 [1)]	0.5	0.75 [1)]	0.93 ± 0.00	9461.43 ± 36.37	18868.93 ± 172.58	
	non-packaging	colored	3937000 [3)]	0.17 [1), 3)]	0.041 [1)]	0.44	0.75 [1)]	0.92 ± 0.01	8292.13 ± 132.86		

References

Blasenbauer, D., Lipp, A.-M., Fellner, J., Tischberger-Aldrian, A., Stipanović, H., & Lederer, J. (2024). Recovery of plastic packaging from mixed municipal solid waste. A case study from Austria. *Waste Management*, *180*, 9–22. https://doi.org/10.1016/j.wasman.2024.02.040

BMK. (2021). *Die Bestandsaufnahme der Abfallwirtschaft in Österreich—Statusbericht 2021 (Referenzjahr 2019)*.

Lipp, A.-M., & Lederer, J. (2025). The circular economy of packaging waste in Austria: An evaluation based on statistical entropy and material flow analysis. *Resources, Conservation and Recycling*, *217*, 108193. https://doi.org/10.1016/j.resconrec.2025.108193
